# Lignin Polyurethane Aerogels: Influence of Solvent on Textural Properties

**DOI:** 10.3390/gels10120827

**Published:** 2024-12-14

**Authors:** Razan Altarabeen, Dmitri Rusakov, Erik Manke, Lara Gibowsky, Baldur Schroeter, Falk Liebner, Irina Smirnova

**Affiliations:** 1 Institute for Thermal Separation Processes, Hamburg University of Technology, 21073 Hamburg, Germany; erik.manke@tuhh.de (E.M.); lara.gibowsky@tuhh.de (L.G.); baldur.schroeter@tuhh.de (B.S.); irina.smirnova@tuhh.de (I.S.); 2 United Nations University Hub on Engineering to Face Climate Change at the Hamburg University of Technology, United Nations University, Institute for Water, Environment and Health (UNU-INWEH), 21073 Hamburg, Germany; 3 Institute of Chemistry of Renewable Resources, University of Natural Resources and Life Sciences, Vienna 3430, Austriafalk.liebner@boku.ac.at (F.L.)

**Keywords:** lignin, biobased polyols, biobased aerogels, green chemistry, sustainable porous materials

## Abstract

This study explores the innovative potential of native lignin as a sustainable biopolyol for synthesizing polyurethane aerogels with variable microstructures, significant specific surface areas, and high mechanical stability. Three types of lignin—Organosolv, Aquasolv, and Soda lignin—were evaluated based on structural characteristics, Klason lignin content, and particle size, with Organosolv lignin being identified as the optimal candidate. The microstructure of lignin polyurethane samples was adjustable by solvent choice: Gelation in DMSO and pyridine, with high affinity to lignin, resulted in dense materials with low specific surface areas, while the use of the low-affinity solvent e.g acetone led to aggregated, macroporous materials due to microphase separation. Microstructural control was achieved by use of DMSO/acetone and pyridine/acetone solvent mixtures, which balanced gelation and phase separation to produce fine, homogeneous, mesoporous materials. Specifically, a 75% DMSO/acetone mixture yielded mechanically stable lignin polyurethane aerogels with a low envelope density of 0.49 g cm^−3^ and a specific surface area of ~300 m^2^ g^−1^. This study demonstrates a versatile approach to tailoring lignin polyurethane aerogels with adjustable textural and mechanical properties by simple adjustment of the solvent composition, highlighting the critical role of solvent–lignin interactions during gelation and offering a pathway to sustainable, high-performance materials.

## 1. Introduction

The pressing challenges of climate change have driven the United Nations to establish several Sustainable Development Goals (SDGs) as guidelines to achieve a more sustainable and equitable world by 2030 [1]. Biorefineries are the cornerstone of a biobased circular economy, transforming biomass into valuable products and sustainable materials, directly contributing to the achievement of SDG 12 and SDG 13. Moreover, biorefineries are essential in paving the way for the broader implementation of a sustainable bioeconomy [2]. Second-generation biorefineries have the potential to meet these SDGs without exacerbating issues like food security. Most research related to biomass upgrading focuses on converting C5 and C6 sugars [3]. However, other biorefinery fractions, such as lignin, offer significant potential for designing new sustainable functional materials, particularly when considering the scale of available raw materials and their final conversion into value-added products [2,4].

Lignin is a complex biopolymer found in the cell walls of plants, where it provides structural support, rigidity, and resistance to decay [5]. It is composed of aromatic phenylpropane units linked by carbon-carbon and ether bonds, making it a highly resilient and chemically diverse material [6]. It is also the second most abundant natural polymer after cellulose, accounting for approximately 20–30% of the dry weight of wood [7]. Despite its availability and large-scale production as a by-product of the pulp and paper industry, lignin is not as widely used as cellulose and is often underutilized [8]. As compared to cellulose, lignin’s unique and complex aromatic molecular structure imparts rigidity and higher thermal stability, making it an attractive source for materials usable in various applications, including electronics, adhesives, and coatings [6,9,10].

This combination of abundance and structural properties makes lignin a generally promising resource for the fabrication of sustainable materials [11]. In particular, in polyurethane chemistry, lignin can enhance sustainability by partially or fully replacing petroleum-based polyols. Hereby, the lignin’s hydroxyl groups can react with diisocyanates to form the essential intra- and intermolecular urethane linkages that make up the structure of polyurethanes [10,12]. Incorporating lignin into established polyurethane products increases not only their biobased content but has also led to performance advantages in certain cases. In most simple cases, lignin can act as a reinforcing filler that contributes to a more cross-linked and structurally robust polymer network which increases the mechanical stability of polyurethane materials [10]. Moreover, lignin enhances flame retardancy by forming a char during combustion, which slows flame spreading and boosts polyurethane fire resistance [13,14,15].

The applicability of lignin-based polyurethanes has been extensively studied in the literature for various uses and sectors, including packaging, construction, automotive, electronics, and biomedical products [10,12]. For instance, Lai et al. (2021) prepared lignin-based waterborne polyurethane emulsions, which exhibited enhanced thermal stability and UV aging resistance, offering a novel biobased polyurethane material [16]. In another study, researchers synthesized lignin-based polyurethane elastomers with enhanced toughness and mechanical strength by crosslinking poly(propylene glycol) with toluene 2,4-diisocyanate, using unmodified lignin as a terminator. An increase in lignin content resulted in higher thermal stability and glass transition temperatures, surpassing the performance of most conventional polyurethane elastomers [17]. However, it is important to highlight that the use and development of lignin-based polyurethanes remains largely limited to the research scale [7].

The most common and extensively studied lignin–polyurethane composite materials are lignin-based polyurethane foams, which are cellular materials with generally low density, formed by polymerization of lignin-derived polyols and isocyanates. The resulting materials exhibit either an open or closed cellular structure, well-suited for different applications such as thermal insulation, cushioning, packaging, and adsorbents [18]. For instance, Ma et al. (2021) fabricated a lignin-based polyurethane foam as a photothermal sorbent that exhibited outstanding adsorption capacity for heavy oil [19]. An approach to enhance the properties of porous lignin polyurethanes even further is to increase their mesopore content by the formation of aerogels. Due to the presence of open, mesoporous networks, aerogels show a combination of high internal specific surface area, good internal mass transport capability, and high overall porosity, leading to a range of new applications [20].

In the first step of lignin-polyurethane aerogel production, lignin reacts with isocyanates in the presence of an anhydrous organic solvent, creating an open-porous, wet organogel. The fluid phase is subsequently removed from the solid network matrix formed through drying techniques, such as supercritical carbon dioxide drying (scCO_2_ drying) [20]. Merillas et al. (2022) synthesized polyurethane foam/polyurethane aerogel composites by incorporating polyurethane aerogels into the foam skeleton. The inclusion of aerogels significantly enhanced the elastic modulus of the foams while reducing the thermal conductivity of the composites to 16.6 mW m^−1^ K^−1^, compared to 40.3 mW m^−1^ K^−1^ for pure polyurethane foams [21]. This example highlights the fact that polyurethane aerogels possess not only significant mesopore-contents but may also feature superior thermal insulating properties compared to traditional polyurethane foams, depending on their density [20,22]. Several studies have highlighted the strong correlation between the envelope density of polyurethane aerogels and their thermal conductivity [22,23,24]. For example, Diascorn et al. (2015) identified an optimal density range for polyurethane aerogels of (0.17–0.19 g cm^−3^) to achieve a minimal thermal conductivity of 0.017 W m^−1^ K^−1^ [22]. Despite their potential, there is a noticeable gap in the literature concerning the exploration of lignin-based polyurethanes as aerogels, since the use of lignin in the fabrication process poses several challenges.

For instance, dissolution behavior and reactivity of lignin are generally difficult to predict and remain poorly understood due to the complex macromolecular structure of lignin which can vary to a large extent with its natural origin as well as the applied extraction (pulping), separation, and purification methods. These variations significantly impact the structural and physical characteristics of lignin, leading to inconsistencies in the properties of lignin-based materials [25,26]. The amphiphilic nature of lignins, along with pulping-related lignin derivatization and significant macromolecular variations caused by condensation reactions or the presence of lignin–carbohydrate complexes, can lead to: (1) poor solubility in various solvents, complicating its processing and impacting the uniformity and mechanical properties of the aerogels produced, and (2) steric hindrance, which may render a certain fraction of hydroxyl groups less accessible to isocyanates, thereby lowering the degree of crosslinking in the final gel [27,28,29].

In this study, we aim at the production of purely lignin-based polyurethane aerogels and will assess how variations in lignin type affect its potential as a biopolyol, as well as how the choice of different solvents influence the microstructural properties of the products. For this reason, three different types of lignin were selected: Organosolv lignin, Aquasolv lignin, and Soda lignin. Their solubility behavior and key characteristics were examined to determine the optimal lignin choice. The role of solvents in minimizing lignin’s steric hindrance during the gelation step was investigated by fabricating lignin polyurethane aerogels in various solvents, including dimethyl sulfoxide (DMSO), pyridine, acetone, and their cosolvent mixtures. The textural and microstructural properties of the resulting lignin polyurethane aerogels were subsequently evaluated to gain a deeper understanding and establish correlations.

## 2. Results and Discussion

Since understanding the molecular structure and properties of lignin is crucial for its utilization as a polyol in the fabrication of lignin-based polyurethane aerogels, different types of lignin were first analyzed with regard to particle size, structural characteristics, and solubility behavior (Section 2.1). Subsequently, the most promising lignin type was used to prepare lignin polyurethane samples, and the properties of the resulting aerogels were described as a function of the solvent used (Section 2.2).

### 2.1. Lignin Characterization

#### 2.1.1. Lignin Particle Size Distribution

**Figure 1:**
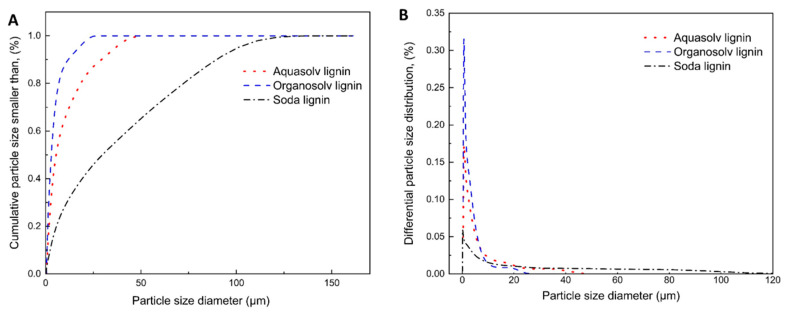
Particle size distributions of different lignins: cumulative (**A**) and differential (**B**).

The particle size of lignin is critical to its behavior and solubility, with smaller particles providing increased surface area, enhancing chemical and physical interactions, and resulting in faster dissolution rates [30]. Lignin particle size was evaluated using cumulative and differential particle size distributions (Figure 1). Aquasolv and Organosolv lignins displayed monomodal particle size distributions, while Soda lignin showed a broad distribution with particle sizes ranging from approx. 0.4 µm to 147 µm. Organosolv lignin had the smallest particle size, with a median (D50) of 2.9 µm and 90% of the particles (D90) being smaller than 11.9 µm.

The particle size of purified lignin powders is primarily influenced by two factors: the extraction or purification process itself and the subsequent recovery/drying method. In extraction, parameters, such as solvent, pH value, and temperature, significantly affect both its structural integrity and particle size [31]. For instance, the Organosolv process employs milder conditions compared to other extraction methods, such as kraft or soda pulping, which helps to preserve the lignin structure to a larger extent while avoiding nonuniform bond cleavage and depolymerization. Furthermore, the oxidative condensation and subsequent agglomeration of lignin to particles does not occur during Organosolv pulping which entails narrower particle size distributions and lower mean particle diameters. This is consistent with our work since the studied Organosolv lignin was found to feature the smallest particle size [31,32]. Aquasolv lignin exhibited a slightly larger mean particle diameter and a narrow particle size distribution. This outcome can be attributed to the recovery method used for Aquasolv lignin, which involves a spray drying technique, providing better control over particle size and morphology [33]. Finally, Soda lignin had the largest and broadest particle size distribution, with a median particle size of 29.7 µm. This larger particle size is likely due to the harsh alkaline conditions used in soda pulping, which promote the recombination of lignin fragments through condensation reactions, leading to the formation of larger macromolecules and their subsequent precipitation [31,34,35].

#### 2.1.2. Structural Characteristics of Lignins

As previously stated, different types of lignins derived from wheat straw and beech wood sources and different purification processes exhibit distinct behaviors due to their unique molecular structures [36]. A summary of these characteristics for the different purified lignin types is provided in Table 1.

Klason lignin analysis can give valuable information about the lignin content of lignocellulosic materials and the purity of lignin samples, respectively, since two-step acid-mediated hydrolysis of oligo- and polysaccharides remove quantitatively physically adhering and chemically bonded “sugars” in terms of mono, oligo, and polysaccharides. After subtraction of the inorganic weight fraction (determined as “ash” content) from the remaining washed (H_2_O) and dried organic residue, a “lignin purity value” is obtained, suitable to assess the applicability of the respective lignin for a wide range of applications. A comparison of the studied Aquasolv and Organosolv lignins originated from different biomasses reveals that the latter have significantly higher purity (Table 1). This is supposedly due to the high silica content of the wheat straw used in the Aquasolv process which can account for up to 90 wt.% of the inorganic fraction [37]. When considering its use as a polyol component in polyurethanes, it is important to note that some of the silica in wheat straw exists as finely dispersed amorphous silica, abundant in surface hydroxyl groups. These hydroxyl groups could react with isocyanates, potentially improving the mechanical performance of the polyurethane.

In the context of the targeted use of lignin as an aromatic polyol component in the manufacture of polyurethane aerogels, the contents of aliphatic and phenolic hydroxyl groups are of particular interest, since they are anchor points for reaction with either flexible (aliphatic) or more rigid (aromatic) di- or polyfunctional isocyanates for three-dimensional crosslinking. Quantification of these aliphatic and phenolic hydroxyl groups by ^31^P-NMR spectroscopy after phosphitylation with the established reagent 2-chloro-4,4,5,5-tetramethyl-1,3,2-dioxaphospholane (TMDP; [38]) revealed a higher content of hydroxyl groups for the Organosolv lignin (5.03 mmol g^−1^) while it was lower for the wheat straw Aquasolv (2.71 mmol g^−1^) and Soda lignins (1.35 mmol g^−1^; Table 1). Considering that a certain part of the hydroxyl groups is typically “trapped” within the three-dimensional lignin macromolecule and is not accessible for isocyanate crosslinking, the Organosolv lignin with the highest content of hydroxyl groups seems particularly attractive for replacing a certain percentage of traditional polyols in polyurethane synthesis.

However, the disadvantage of wheat straw Soda lignin in terms of having the lowest hydroxyl group contents among the tested lignins is compensated by its relatively high weight-average molecular weight (6.2 kDa, Table 1). Compared to the beech wood Organosolv lignin (Mw 3.7 kDa), the higher weight-average molecular weight is assumed to provide a better network-expanding effect upon reaction with isocyanates as discussed in previous works by Ghorbani et al. or Tejado et al. for lignin-phenol-formaldehyde composites [39,40].

#### 2.1.3. Lignin Solubility

Molecular dispersing dissolution of the involved reactants is a critical factor for the crosslinking by polyaddition reaction between isocyanates and polyols, as it decides the uniformity of the gels obtained in an advanced state of network formation [41,42,43]. Adequate solubility facilitates necessary interactions e.g., cross-linking and aggregation of higher-molecular polyurethanes to form respective gel networks. Additionally, solubility influences both the rate and mechanism of gelation while diisocyanates readily dissolve in anhydrous organic solvents, this is not always the case with lignin due to its amphiphilicity, variation in macromolecular characteristics, functional group contents, and specific spatial arrangement and self-assembly in those solvents. Due to the complexity of these interfering factors, the exact mechanisms behind dissolution, solvation, and hydrodynamic arrangement remain still largely unclear [44].

In a fundamental approach, the solubility behavior of polymers can be generally predicted using a combination of Hildebrand and Hansen solubility parameters [45]. In simple terms, two materials are likely to be miscible if their Hildebrand or Hansen parameters are similar. The Hildebrand parameter provides a single-value measure of cohesive energy density, which is particularly useful for non-polar and slightly polar polymers [46]. Hansen parameters break down solubility into specific interaction components—dispersion, polar, and hydrogen bonding forces [46]. By integrating both parameters, a more comprehensive understanding of complex systems is obtained, accounting for overall cohesion (Hildebrand) and specific molecular interactions (Hansen) which is especially useful for polymers and multi-component systems.

Publicly assessable Hildebrand and Hansen parameter values for various solvents are summarized in Table S1 [45,46,47,48]. A study by Schuerch (1952) tested various solvents and demonstrated that lignin dissolves effectively in solvents with a Hildebrand solubility parameter (δ) of around 22.5 MPa ^1/2^, indicating that this parameter is optimal for lignin solubility [47,49,50]. Consequently, MEK, acetone, ethanol, and DMSO are considered optimal candidates for dissolving lignin. Among the different interaction forces, hydrogen bonding has been identified as the most favorable interaction for enhancing solubility, although very high hydrogen bonding causes lignin molecules to associate or aggregate with each other, thereby reducing solubility [45].

**Figure 2:**
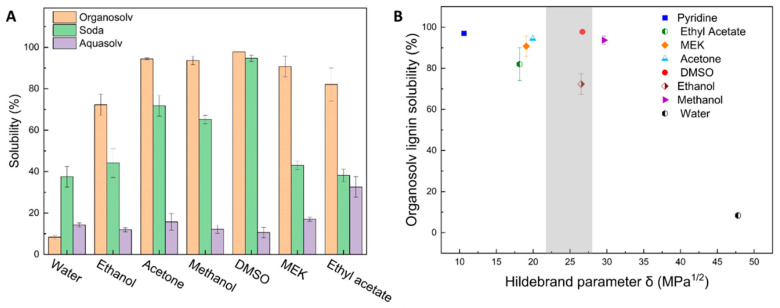
(**A**) Solubility of Aquasolv, Soda, and Organosolv lignin in different solvents at a solute concentration of 3 wt.%, (**B**) Organosolv lignin solubility as a function of Hildebrand Parameter, The grey-shaded area highlights the optimal Hildebrand solubility parameter range (δ) for lignin solubilization.

However, differences between lignin types on the molecular level result in varying solubility in the same solvent [44]. That being said, conclusions drawn from Hildebrand and Hansen solubility parameters may not always be applicable across different lignin types and solubility parameters alone are not sufficient to predict solubility behavior [49]. Therefore, the solubility of various lignins was experimentally evaluated in different solvents (water, ethanol, acetone, DMSO, MEK, and ethyl acetate) by dissolving a constant amount of lignin (3 wt.%) in 30 mL of solvent (Figure 2A). Solubility at different weight concentrations can be found in Figure S1.

As expected, the three types of lignin displayed different solubilities. Organosolv lignin demonstrated the highest solubility in organic solvents, ranging from 72% to 97%, with the highest solubility observed in DMSO. Conversely, the lowest solubility was experimentally observed in water, similar to all other lignin types (Figure 2A). This behavior is expected due to the significant differences in the solubility parameters of lignin and water [45]. This outcome is consistent with Organsolv lignin’s properties, as the Organosolv pulping process produces high-purity lignin with a low molecular weight, characterized by a Klason content of 89.5% and a molecular weight of 3.7 kD, which contributes to enhanced solubility [51]. These results offer further insights when compared to Hildebrand parameters (Figure 2B): solvents with solubility parameters close to the previously reported optimal value of 22.5 MPa^1/2^ (such as DMSO and ethanol) exhibit different solvation abilities for Organosolv lignin, with DMSO demonstrating the highest solubility.

In contrast, Soda lignin displayed a wide range of solubility, from 38% to 94%, with the highest solubility observed in DMSO. This behavior is due to DMSO’s ability to form hydrogen bonds with lignin, effectively disrupting intermolecular interactions, particularly hydrogen bonds within the lignin structure. This disruption aids in breaking down the complex lignin network, thereby enhancing its solubility [45]. On the other hand, the lowest solubility in organic solvents was observed in ethyl acetate, which has lower polarity and limited hydrogen bonding capacity, making it a poor solvent for dissolving Soda lignin [52].

Aquasolv lignin, despite its small particle size and intermediate molecular weight, demonstrated poor solubility with slightly better solubility in ethyl acetate. Due to the limited research on Aquasolv lignin, establishing a direct correlation is challenging [53].

The lignin solubility results were consistent with previous findings on structural characteristics (Section 2.1.2), further confirming the influence of molecular weight and particle size on lignin solubility. Overall, Organosolv lignin exhibits superior characteristics, including favorable particle size distribution for lignin’s dissolution, higher solubility in organic solvents, and greater hydroxyl group content. Consequently, Organosolv lignin was selected for the fabrication of lignin-based polyurethane aerogels in this study.

### 2.2. Textural Properties

**Figure 3:**
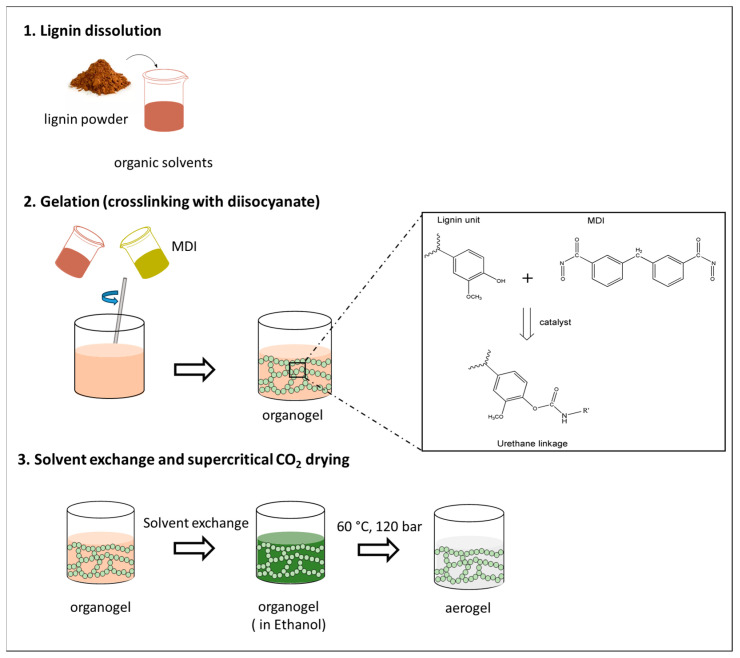
Schematic representation of the synthesis process and gelation of lignin polyurethane aerogels.

The gelation process was performed via sol–gel crosslinking (by polyaddition), in which the transition of a sol into a gel-like network (gel) occurs through the following chemical reaction: lignin acts as the polyol, providing hydroxyl groups that react with isocyanate functional groups to form urethane linkages (Figure 3), the chemical reaction has been extensively explained in previous studies [12,29,54].

#### 2.2.1. Variation of Pure Solvents

Gelation was performed using three different solvents—DMSO, pyridine, and acetone—each with varying solvation powers and lignin–solvent interactions, at a lignin-to-solvent ratio of 8 wt.% (Figure 3).

DMSO was selected for its strong hydrogen-bonding capabilities, offering high affinity to lignin and near-complete solubility for Organosolv lignin (~100%) (Section 2.1.3) [50,55]. Pyridine was chosen as the second solvent due to its similar solubility (~100%) for Organosolv lignin, supported by preliminary testing. High solubility in pyridine is primarily driven by acid-base interactions between the solvent and the phenolic groups in lignin [55], which are not considered in Hildebrand’s theory [45,55]. In contrast to former solvents, acetone exhibits lower and weaker hydrogen-bonding capabilities compared to DMSO, resulting overall in a slightly reduced solubility for lignin (~94%) [55].

**Figure 4:**
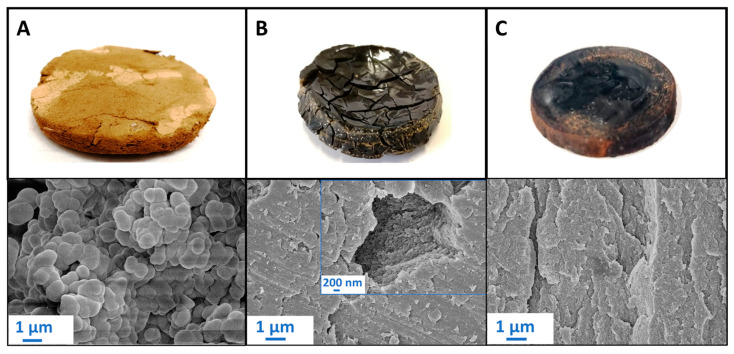
Organosolv lignin polyurethane samples produced in acetone (**A**), DMSO (**B**) and pyridine (**C**).

The addition of MDI as a crosslinker led to the gelation of lignin, with a pronounced influence of the solvent choice on the gelation time (Table 2), as well as the visible appearance and textural properties of the resulting samples (Table 2, Figure 4). The apparent change in color in the samples from pale yellow to dark brown could be attributed to the different solvent-driven interactions with lignin which induce changes in chromophoric systems in lignin thus altering the light absorption properties of lignin and its color [56].

Gelation in acetone was the slowest one and resulted in a brittle, dusty material (Figure 4A). While further processing resulted in comparably low shrinkage and a low-density material with high overall porosity, no measurable specific surface area was detected, suggesting it is purely macroporous in nature. Scanning Electron Microscopy (SEM) images of the inner structure reveal the formation of larger aggregates (approx. 1 µm in size), which form a continuous, macroporous system.

We suggest, that in the case of the “lower-affinity” solvent such as acetone, differences in reagent solubility led to de-mixing, resulting in microscale phase separation during gelation: this combination of microphase separation and delayed structure formation due to the slow kinetics then led to excessive coarsening of the phase-separated, micrometer-sized particles [57,58]. Similar morphologies have been observed in other studies involving phase-separated polymer strands, described as a “string of pearls” or “sponge-like” structure, in Resorcinol-Formaldehyde gels and poly(TMPTA-VTES) coatings [59,60,61].

The use of DMSO resulted in significantly faster gelation time, whereas the use of both pyridine and DMSO led to higher shrinkage and overall stiffer and less porous materials with elevated specific surface areas (Table 2).

SEM images show in both cases the absence of macropores and the presence of densely packed, partly mesoporous lignin layers (Figure 4B,C) which contribute most probably to the slight increase in surface area. In contrast to acetone, solvents with higher lignin affinity prevent microphase separation, leading to faster gelation due to enhanced solvent-solute interactions. Since lignin remains dissolved, crosslinking promotes the formation of small primary particles. These particles grow and interconnect, forming a three-dimensional network. As the structure approaches the gel point, early structural formation occurs, resulting in a fine, dense microstructure [42,57,58].

While DMSO has proven to be the most suitable single solvent to facilitate the formation of mesopores, the main challenge is the significant volumetric shrinkage of 80%, resulting in a highly dense material. This shrinkage is most likely to be attributed to DMSO’s strong affinity for lignin, allowing it to penetrate not only into the pores of the lignin-polyurethane gel matrix but also into the pore walls, which are built up by lignin’s branched, three-dimensional structure. During supercritical CO_2_ drying, DMSO is extracted from the pores and only partly (see TGA analysis in Section 2.2.2) from the internal lignin walls. This process likely induces mechanical stress on the solid matrix, causing it to collapse during drying, leading to substantial shrinkage and a reduced volumetric yield of the final material [62].

**Figure 5:**
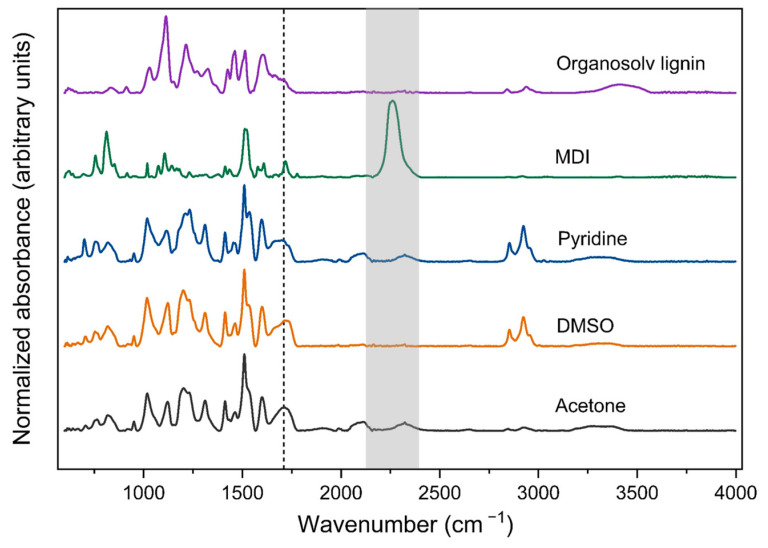
FTIR spectra of Organosolv lignin polyurethane samples fabricated in different solvents and their educts: Organosolv lignin and MDI.

Previous studies have shown that solvents like DMSO, which exhibit a high affinity for the biopolymer matrix, may serve as excellent gelation solvents and even facilitate gentle solvent exchange. However, they pose challenges during supercritical CO_2_ drying [62]. This statement is supported by FTIR results (Figure 5), which show DMSO to be still present in the samples after supercritical CO_2_ drying. This could also lead to evaporative drying of the residual solvent traces e.g., during the degassing step (before BET measurements), causing additional pore collapse after the supercritical CO_2_ drying process.

Furthermore, FTIR analysis of the starting materials and samples (Figure 5) confirmed successful crosslinking between lignin and diisocyanate (MDI), as well as the formation of urethane bonds in all samples. This is evident from the disappearance/reduction of the intensity of the primary absorbance peak at 2262 cm^−1^ in the MDI spectrum, corresponding to isocyanate groups [63]. The emergence of a broad peak at 1710 cm^−1^, attributed to the stretching vibration of urethane bonds, verified the crosslinking between Organosolv lignin and MDI [20]. A small peak around 2262 cm^−1^ being present in acetone and pyridine-derived samples shows that a small amount of free surface N=C=O groups is still present in the samples, and indicates that MDI has not completely reacted in these cases. The small peaks in the region of 2000–2300 cm^−1^ might indicate accordingly the presence of additional MDI-based byproducts (e.g., derived from incomplete reacted MDI). Peaks at 2850 and 2920 cm^−1^ are attributed to aliphatic symmetric and asymmetric -CH_2_ stretching vibrations [20,64]. The shift of the broad signal from ~ 3250–3600 cm^−1^ (OH-stretching) in pure lignin to lower wavenumbers (~3100–3400 cm^−1^) in the case of crosslinked samples is most likely to be attributed to the presence of urethane N-H groups [65]. 

Even though the exact definition of the term “aerogel” is still under discussion in the scientific community, essential characteristics from a material point of view are as follows: a high porosity, significant specific surface area (greater than ~100 m^2^ g^−1^), and an open, three-dimensional mesoporous structure. These criteria were not met when using pure DMSO and pyridine, which showed too high densities and non-sufficient mesoporosity.

In contrast, mainly macroporous materials (e.g., in many cases derived from freeze-drying) without a significant specific surface area can be described as sponges or open foams [66,67]. This description matches our case with the aggregated, macroporous sample produced in acetone.

To address the challenges posed by both microphase separation in lower-affinity solvents (e.g., acetone) and high volumetric shrinkage in higher-affinity solvents (e.g., DMSO and pyridine), solvent mixtures were used as gelation solvents instead of relying on a single solvent.

#### 2.2.2. Solvent Mixtures

**Figure 6:**
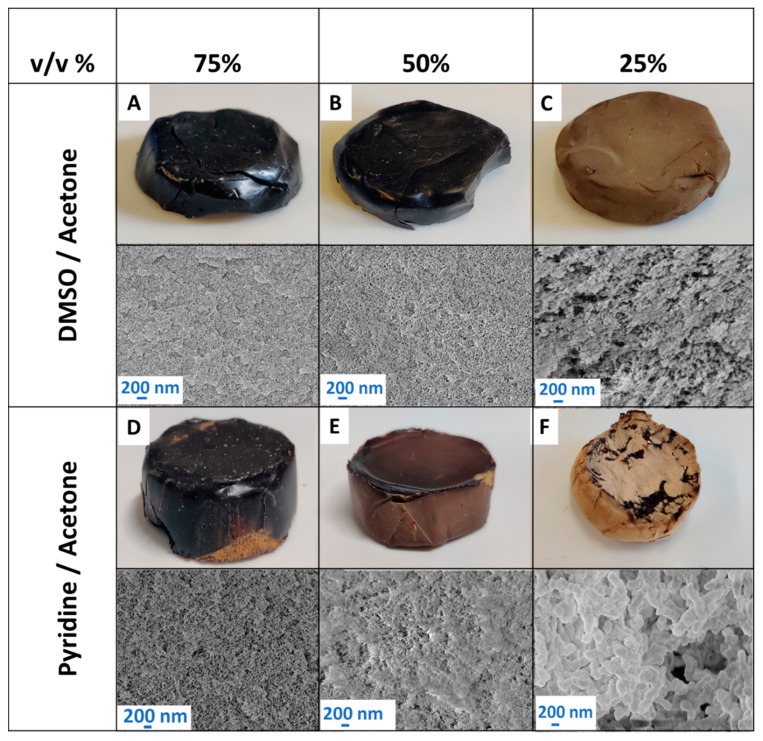
Organosolv lignin polyurethane aerogel produced in solvent mixtures: DMSO/acetone (DA) in the upper row: DA25 (**A**), DA50 (**B**), and DA75 (**C**). Pyridine/acetone in the lower row: PA25 (**D**), PA50 (**E**) and PA75 (**F**).

Gelation was performed using solvent mixtures of DMSO/acetone (DA) and pyridine/acetone (PA) at 25%, 50%, 75%, and 100% DMSO/pyridine by volume. The variations in optical appearance and microstructures in these samples (Figure 6) indicate competition between domain coarsening (due to microphase separation) and structural formation (via gelation).

Consistent with observations from pure solvents (see Section 2.2.1), gelation time (t__gel_) depends on the solvent ratio and type, with faster gelation in DMSO than in pyridine-containing mixtures. Increasing the acetone ratio lengthens gelation times, showing a strong linear correlation (R^2^ = 0.98, Figure 6A). Changes in microstructural and textural properties of samples are subsequently discussed from low to high DMSO/pyridine content.

**Figure 7:**
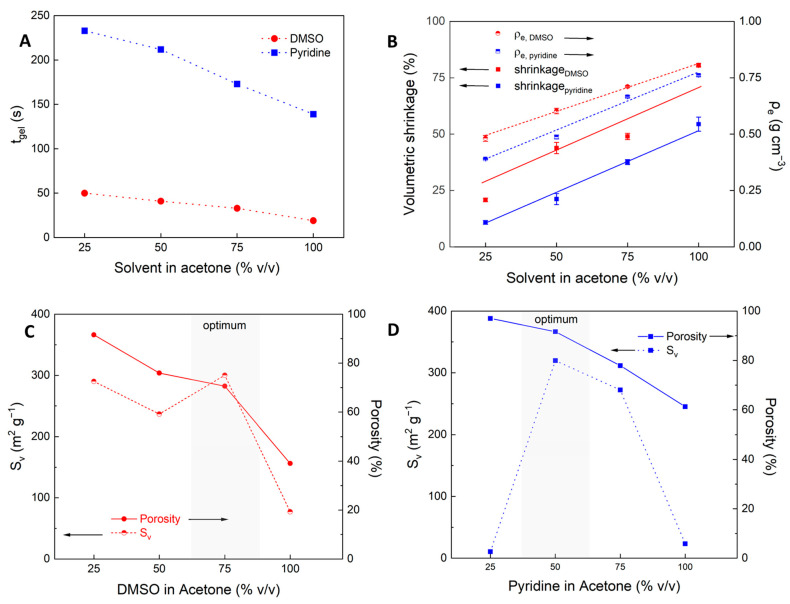
Various properties of DMSO/acetone- and pyridine/acetone-based polyurethane samples in dependence of solvent mixtures: (**A**) gelation time, (**B**) envelope density and volumetric shrinkage, (**C**) Specific surface area and total porosity for DMSO/acetone, and (**D**) for pyridine/acetone mixtures. Lines are drawn to guide the eye whereas the grey shaded areas indicate the optimal solvent composition ranges for maximizing specific surface area (*S*_v_) within the overall porosity of the samples.

In DA/PA-25 solvent mixtures, reagents de-mix relatively early, while structural formation occurs at the end of phase separation due to slow kinetics. This results in extensive coarsening, with larger building blocks and lower specific surface areas. A micro-aggregated morphology in PA25, similar to that in pure acetone samples, is observed. It is although notable, that the presence of pyridine led to finer aggregates (~200–400 nm as compared to ~500–1000 nm, see Figure 6F and Figure 4A). In contrast, for DA25, the structural formation occurs at an earlier stage than in PA25, as evidenced by the gelation kinetics (Figure 7A).

In DA50 and PA50 mixtures, structural formation and phase separation seem to occur rather simultaneously. At intermediate gelation stages, primary particles grow and interconnect, leading to a broader pore size distribution and a fixed, interconnected network morphology. The timing of stabilization significantly influences the final microstructure, with PA50 showing earlier microscale phase separation and slower gelation than DA mixtures at the same composition.

As solvent mixtures shift to higher DMSO/pyridine concentrations (PA75 and DA75), faster gelation leads to earlier structural formation, producing finer microstructures and improved mesoporosity (Figure 6A,D). However, moving further towards pure solvents results in too early structural formation, and denser, less mesoporous materials (see Section 2.2.1, Figure 4).

This trend is linked to how solvents impact the reactivity of lignin, particularly by influencing its conformational flexibility [68]. In high-affinity solvents like DMSO, lignin adopts more extended conformations, facilitating the intermolecular interactions necessary for crosslinking during gelation. Conversely, in low-affinity solvents like acetone, lignin adopts more compact conformations that hinder crosslinking and may contribute to steric hindrance [69].

The clear trend of the influence of solvent mixtures on gelation time (t_gel_) (Figure 7A), was also found for the textural properties. In both cases, the shift of solvent mixtures to higher affinity was accompanied by an increase in volumetric shrinkage and subsequently an increase in envelope density for both DMSO and pyridine (Figure 7B). The measured density values, specifically for samples with a significant specific surface area exceeding ~100 m^2^ g^−1^, are within the range of 0.21–0.49 g cm^−3^ for DMSO-based samples and 0.21–0.38 g cm^−3^ for pyridine-based samples. It should be noted that these values are considered relatively high and require further optimization to be considered suitable for thermal insulation applications [22,24].

Regarding the microstructure morphology, low-affinity solvents like acetone produced micro-sized aggregates, which build up foam-like macroporous materials with no detectable specific surface area, resulting in overall low density and fragile structures (Section 2.2.1). Shifting to higher concentrations of high-affinity solvents DMSO and pyridine decreased the overall porosity (decrease of macroporosity) while enhancing the mesoporous content in intermediate solvent mixtures, as reflected by an increase of specific surface area (Figure 7C,D). This shift suggests a transition from macropores to a finer structure with increased mesoporosity and is consistent with SEM images, showing homogenously distributed mesopores with approximate pore diameters of ~<100 nm visible in samples produced at DA/PA ≥ 50%.

Notably, pyridine-based samples showed a transition of the pore structure at relatively low pyridine content: while sample PA25 exhibits a still and aggregated structure, a max. of S_V_ = 319 m^2^ g^−1^ and a change of the structure is already observed after an increase to 50% (PA50). However, a further increase in the pyridine volume fraction reduces both the overall porosity and specific surface area. In the case of DMSO, an already low content of 25% leads to a mesoporous material with a significant specific surface area. Increasing the DMSO fraction from 50% to 75% (DA75) slightly decreased the overall porosity but significantly enhanced the specific surface area of up to ~300 m^2^ g^−1^, indicating (a) a further increase of mesopore volume by the transition of macropores to mesopores, or (b) a reduction in mesopore diameter.

Summarizing, results clearly show that the application of solvent mixtures led to low-density materials with sufficient and open mesoporosity, which can be regarded as aerogels. However, the question arises as to whether the change in the different samples’ microstructures is purely induced by lignin-conformation and phase-separation behavior, or if the chemical composition (amount of crosslinking) was also affected by the adjustment of solvent mixture. Additional analytics (FTIR, TGA) were therefore carried out for samples produced in DMSO-mixtures.

**Figure 8:**
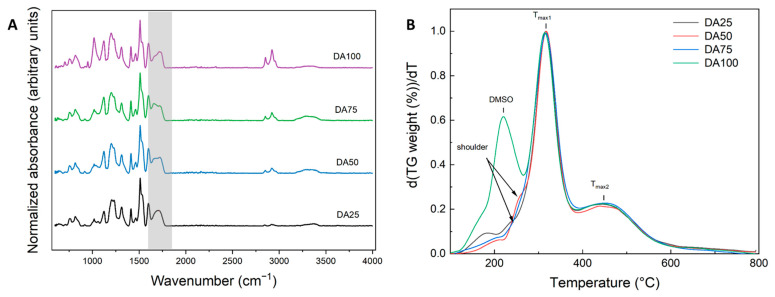
(**A**) FTIR spectra and (**B**) Derivative Thermogravimetric Analysis (DTG) of the Organosolv lignin polyurethane aerogels produced using solvent mixtures of DMSO/Acetone (DA).

The successful crosslinking between lignin and MDI and the formation of urethane bonds was assessed via FTIR analysis (Figure 8A). The spectra of samples prepared in solvent mixtures were found to be similar to those prepared using pure solvents, where the formation of urethane bonds was indicated by a broad peak at ~1710 cm^−1^ and the absence of free isocyanate groups was confirmed by the lack of a signal at 2262 cm^−1^ [20,70]. No significant differences were found for the signal urethane-bond intensities to lignin-related signals (e.g., between 970–1650 cm^−1^) in between samples, which indicates an equal amount of crosslinking in all cases.

The thermogravimetric (TGA, Figure S2B) and differential thermogravimetric curves (DTGA, Figure 8B) show that all samples follow the same decomposition mechanism. Differences in the course of the DA100 sample can be attributed to the presence of residual DMSO (estimated DMSO weight ratio regarding solid matrix based on TGA-data: ~20 wt.%) and verifies that it was not possible to remove DMSO with pure ethanol-based solvent exchange and supercritical CO_2_ drying from the lignin-matrix. It is suggested that only the “free” DMSO filling the pores was washed out during the solvent exchange, while the DMSO directly associated with the lignin matrix stayed embedded in the pore matrix (leading in this case to a “misleading” value of 97% ethanol in the liquid phase during the solvent exchange, which does not account for the DMSO present as associated directly to lignin). It is also notable that this result was only obtained in the case of pure DMSO as a solvent, while no significant leftovers of DMSO were found in samples produced in DMSO/acetone solvent mixtures. While understanding the particular reason for this behavior on a molecular level is out of the scope of this study, molecular-scale lignin simulations have shown, that (1) maximum lignin conformational expansion was found for DMSO as compared to many organic solvents (including acetone) (2) DMSO has a particular slow diffusion coefficient in lignin [69]. Different solvent mixtures (e.g., additional presence of acetone) may significantly influence lignin conformations/extension as well as resulting solvent diffusion rates [69]. In our case, the results indicate that the presence of acetone as a co-solvent in the ternary mixture significantly enhances the removal of DMSO during solvent exchange.

The thermal decomposition of the lignin–PU matrix itself is generally complex, with many chemical reactions occurring simultaneously or in quick follow-up steps (e.g., breaking of the main chain, decomposition of side fragments accompanied by formation of char residues and volatile components).

It is so far accepted that polyurethanes undergo thermal degradation in two to three main steps, whereas the composition of the products and exact temperature ranges depend on the individual polyurethane (PU)-structure [71,72]. The first step (temperature range of ~200–270 °C) involves the breakage of polyurethane linkages, which follows three mechanisms: dissociation of the urethane-bond resulting in regeneration of the isocyanate and polyol components, 2. dissociation of the urethane-bond under formation of a primary amine, an olefin and CO_2_, and 3. dissociation of the urethane-bond under formation of a secondary amine and CO_2_ [71,73].

The overall mass loss in this step is expected to be relatively low since degradation products are not volatile (except for CO_2_). Step 2 then involves the further degradation reaction, foremost the degradation of the polyol (in our case: lignin) which was regenerated in step 1. Analysis of the pure lignin starting material used in this work shows, that lignin decomposition covers a broad temperature range (onset temperature of t_onset_~150 °C, maximum decomposition rate at 349 °C, followed by a more sustained decomposition at temperatures > 440 °C, see Figure S3A). This broad range is expected due to the numerous oxygen-based functional groups present in lignin with different thermal stabilities [74]. A third decomposition step (T > 380 °C) was e.g., observed in MDI crosslinked Kraft-lignin-PU composites and related to structures formed after the second decomposition step or to further cleavage of the diisocyanates aromatic structure [71,75]. The occurrence of additional decomposition in the temperature range between 350 and 510 °C is indeed also reported in the pyrolysis of pure MDI [76].

While the DTGA patterns observed in this work for lignin-PU aerogels match generally aforementioned descriptions, a clear distinction between steps 1 (breakage of urethane bond) and step 2 (lignin decomposition) is not possible, since individual profiles overlap and result in a representation with slight shoulders. The main DTGA signal with the first-rate maximum at T_max1_ = 349 °C covers the largest mass loss (~67%), while the second signal (t > 390 °C, T_max2_ = 448 °C) is most probably associated with the decomposition of MDI, which was regenerated during cleavage of the urethane-bond [76]. Following this argumentation, ratios of signals T_max1_ and T_max2_ are representative for the lignin-to-isocyanate ratio, which is indicated as being constant throughout all samples. Also, no significant changes in overall mass losses (54.9 ± 0.86%) of samples DA25–DA75 were detected. Notably, TGA analysis of samples produced in pyridine (exemplary carried out for PA25 and PA50 samples) showed no significant differences as compared to samples produced in DMSO mixtures, which indicates that the chemical composition of materials is independent of the solvent choice (see Figure S3A,B).

Overall, TGA results are consistent with FTIR analysis: both methods show that the amount of crosslinking is largely similar in all samples. Variation of solvent mixtures is therefore suited to induce pronounced microstructural changes, without interfering with the chemical nature of the produced materials.

### 2.3. Mechanical Properties

**Figure 9:**
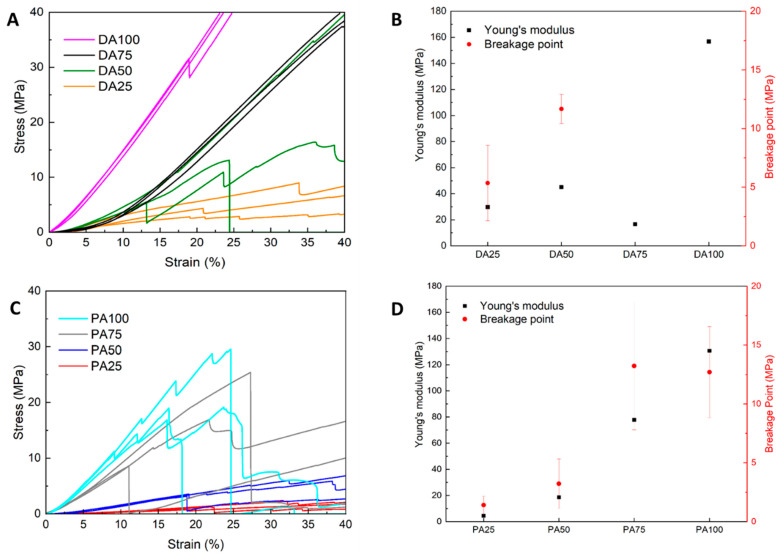
Mechanical property evaluation of samples showing stress-strain curve and Young’s modulus (E) for both DMSO/Acetone samples (**A**,**B**) and pyridine/acetone samples (**C**,**D**). The “breakage point” of the samples was identified from the strain–stress curves at the onset of the first observable failure. Samples were measured in triplicates (*n* = 3).

Mechanical properties were evaluated using uniaxial compression with a strain limit of 10% (Figure 9A,C). Hereby, the beginning of the stress-strain curve was not ideally linear, which was attributed to the slightly uneven surface of the samples (see e.g., sample images in Figure 6). This region was therefore not included in the evaluation. Instead, the subsequently following linear region was used to estimate the Young’s modulus (E) individually for each sample. A minimum R^2^ value of 0.995 was used as the criterion to calculate the elastic modulus (for raw data we refer to Figures S4 and S5, Table S2). The “breakage point” was determined from the stress-strain curves as the onset of the first observable failure.

The samples exhibited varied mechanical behavior, as shown in the stress-strain curves (Figure 9A,C). The acetone-based sample was excluded due to fragility. Samples with the highest acetone concentrations, PA25 and DA25, exhibited high elasticity but low mechanical stability, attributed to their sponge-like morphology [61]. In contrast, pure solvent samples PA100 and DA100 showed low elasticity but high compressive strength, with DA100 demonstrating the highest mechanical resilience under load, with no observable breakage even at the maximum load of 500 N.

Intermediate samples, PA50 and DA50, exhibited distinct mechanical properties. Both samples displayed relatively lower elasticity mainly due to the transition from a micro-dominated structure in PA25/DA25 to a mesoporous-dominated structure in PA50/DA50, where increased porosity contributed to decreased elasticity [77,78]. PA50 demonstrated higher elasticity, whereas DA50 exhibited enhanced mechanical stability.

Increasing solvent affinity led to distinct mechanical behaviors between pyridine-based and DMSO-based samples, as reflected in their microstructure (Section 2.2.2). PA75, with lower porosity and specific surface area (Figure 6D), exhibited reduced elasticity but higher compressive strength, primarily due to increased density. In contrast, DA75 had a higher specific surface area, indicating a transition from macropores to mesopores (Figure 6C). This transition enhanced elasticity and structural integrity under load, resulting in a more mechanically robust material.

Generally, synthetic polymer aerogels exhibit a broad range of Young’s modulus, from 0.1 to 1000 MPa [79]. A study on the mechanical properties of polyurethane aerogels synthesized with different triisocyanates and aromatic polyols reported that Tris(hydroxyphenyl)ethane-based polyurethane aerogels exhibit a wide range of Young’s modulus between 1.0 and 363 MPa under quasi-static compression testing, comparable to the values observed in this work [80] and thus similar to those proposed by Young´s modulus from this work. Flexibility is a desirable characteristic for thermal insulation applications where polyurethane aerogels are of interest.

## 3. Conclusions

This study highlights the potential of lignin to be used as a bio-polyol source to produce sustainable polyurethane aerogels and underscores the critical role of lignin type and lignin–solvent interactions in the production process. Variations in lignin’s structure, influenced by biomass source and extraction or purification methods, result in unique and complex characteristics that challenge traditional solubility predictions, such as those based on Hildebrand and Hansen parameters. Among the three lignin types studied, Organosolv lignin was identified as the optimal choice for use as a biopolyol, owing to its lower molecular weight, higher density of reactive functional groups, and superior solubility in organic solvents.

Our findings revealed that solvent–lignin interactions significantly affect gelation and the resulting lignin polyurethane aerogel microstructures. Specifically, hydrogen-bonding interactions (as seen with DMSO) had a more pronounced impact than acid-base interactions (as seen with pyridine), promoting faster gelation. However, using single solvents alone did not yield materials that met the criteria for being defined as aerogels. To overcome this, we explored solvent mixtures, such as DMSO/acetone and pyridine/acetone, which successfully balanced gelation and phase separation. This approach reduced shrinkage, led to the formation of aerogels with enhanced mesoporosity (high specific surface areas), and improved mechanical stability.

Our study also confirmed that chemical crosslinking remained consistent across different solvent mixtures, while mechanical testing demonstrated how varying solvent ratios influence the elasticity and strength of the aerogels. These insights emphasize the importance of optimizing lignin–solvent interactions to tailor aerogel properties without altering their chemical composition.

While the results presented deepen the understanding of lignin aerogel production, several questions remain open. Future research should aim to uncover the precise mechanisms by which different solvents influence lignin conformation and gelation behavior. Exploring the effects of alternative solvents and crosslinkers, as well as ensuring the reproducibility of aerogel properties (considering lignin’s natural variability) will be crucial for advancing these materials. Moreover, this deeper understanding should be applied to reducing the envelope density of lignin polyurethane aerogels, thereby enhancing their potential use as thermal insulation materials, which could contribute to SDG 11. Continued optimization efforts will pave the way for the broader adoption of lignin-based polyurethane aerogels in high-performance and sustainable applications.

## 4. Materials and Methods

### 4.1. Materials

Lignin fractions were all kindly provided by different sources: Aquasolv lignin (AS) was isolated from wheat straw via a hydrothermal treatment process at the Hamburg University of Technology, as described in detail elsewhere [81]. Organsolv lignin (OS) was produced from beech wood through an ethanol pulping method at Fraunhofer CBP [32]. Soda lignin was purified using a patented acid extraction technique developed by Tanovis AG [81]. Lupranate M20 isocyanate was provided by BASF Polyurethanes GmbH (Lemfoerde, Germany). Solvents (acetone, ethanol, methanol, MEK, ethyl acetate, DMSO, and pyridine) were purchased from Carl Roth GmbH & Co. KG (Karlsruhe, Germany).

### 4.2. Methods

#### 4.2.1. Lignin Characterization Methods

Particle size analysis: measurements were conducted using the LS 13 320 Laser Diffraction Analyzer (Beckman Coulter, Krefeld Germany), employing laser diffraction and light scattering techniques based on either Fraunhofer or Mie theory. The instrument also incorporated Polarization Intensity Differential Scattering (PIDS) technology for the identification of submicron particles, with a measurement range spanning from 0.4 to 2000 μm.

Klason lignin content (according to TAPPI norm T222 om-98)*:* 200 mg of lignin sample was treated under shaking with 6 mL of concentrated sulfuric acid (72 w%, Merck, Darmstadt, Germany) at 30 °C for 60 min. Simultaneously, the dry matter content of the samples (50 mg, two replicates) was determined (105 °C, 24 h). For completion of the hydrolytic step, the reaction mixture was diluted with 84 mL of deionized water (to adjust the H_2_SO_4_ concentration to 4.8 w%) and subsequently autoclaved at 120 °C for 60 min. After cooling to room temperature, the mixture was filtered through a glass frit (3G, 50 mL, 40 mm diameter, Duran, Schott, Mainz, Germany) additionally equipped with a glass fiber filter (GF 92, Schleicher and Schuell, Dassel, Germany) and the filter residue was washed five times with boiling water. The glass frit including the glass fiber filter and filter residue was then dried at 105 °C for 24 h and subsequently cooled to room temperature in a desiccator. The dry mass of lignin in the original sample was calculated from the dry mass of the filter residue and the separately determined dry matter content of the samples.

Solubility tests: tests were performed on all three types of lignin using the same solvent at mass concentrations of 1%, 3%, and 5%. Lignin was added to a sample vessel containing the solvent, shaken vigorously, and then placed in a shaking basin for one hour. Following this, the samples were centrifuged at 4500 rpm for 10 min to separate the undissolved lignin. After centrifugation, the supernatant containing the dissolved lignin was carefully decanted, and the precipitated lignin was dried on an aluminum tray at 100 °C for 24 h. Once dried, the lignin was weighed to determine the amount of undissolved material.

Functional groups analysis (according to Korntner et al. 2015 [38]): 25–30 mg of vacuum-dried lignin was fully dissolved in 700 µL of a mixture of dry CDCl3 and pyridine (1:1.6) by short-time shaking. Subsequently, 200 μL of a stock solution containing the internal standard N-hydroxy-5-norbornene-2,3-dicarboxylic acid imide (e-HNDI, 20 mM) and the NMR relaxation reagent chromium acetylacetonate (Cr(acac)3) was added (5 mg mL^−1^) under continued shaking. After sealing the vial under nitrogen, 100 μL of the phosphitylation reagent 2-chloro-4,4,5,5-tetramethyl-1,3,2-dioxaphospholane (TMDP) was injected through the septum to avoid hydrolysis of the phosphitylation reagent. After a reaction time of 60 min, an aliquot of the mixture was transferred into an NMR tube.

Quantification of aliphatic and phenolic hydroxyl groups after the above phosphitylation was accomplished by 31P NMR spectroscopy using a Bruker Avance II 400 instrument (31P resonance at 162 MHz, Bruker Corporation, Billerica, MA, USA) with a 5 mm N2-cooled broadband observe probe head equipped with z-gradient at room temperature. Data were collected with 64 k data points and apodized with an exponential window function (lb = 5) before FID as described elsewhere [82].

Accelerated Solvent Extraction: Approximately 2 grams of lignin were mixed with 10 g of pre-purified sand and loaded into 60 mL stainless steel cells for subsequent extraction with 150 mL of the respective solvent using a Dionex ASE350 system (Thermo Fisher Scientific Inc., Waltham, MA, USA) (50 °C, 1 h, 10–12 MPa extraction pressure). The extraction was performed four times with identical volumes of solvent for each sample. Each extract was collected in separate bottles to estimate the extraction efficiency. After that, the four extracts per lignin sample and solvent type were merged, and the solvent was evaporated using Büchi Rotavapor equipment (Büchi Labortechnik AG, Flawil, Switzerland)**.** The solid residue was finally dried in an oven overnight at 80 °C.

Molecular weight characterization by size exclusion chromatography (SEC, Zinovyev et al. 2018 [83]): 5–10 mg of lignin was mixed with 1 mL of DMSO/LiBr (0.5% *w*/*v*) and filtered through a 0.45 µm PTFE filter before analysis. SEC analysis of the above prepared samples was performed using the following equipment: Ultimate 3000 autosampler, column oven, UV detector (all Thermo Fisher Scientific Inc., Waltham, MA, USA), Dionex HPLC Pump Series P580 (Dionex Softron GmbH, Germering, Germany), Dawn HELEOS I multi-angle laser light scattering detectors with lasers operating at either 658 or 785 nm, and an Optilab T-rEX differential refractive index detector, λ = 633 nm (all Wyatt Technology, Santa Barbara, CA, USA). Both MALS detectors were equipped with 18 photodiodes at different measuring angles, with narrow band pass filters (±10 nm for the respective wavelength used, installed on every second photodiode). The separation was performed with an Agilent PolarGel M guard column (7.5 mm × 50 mm) and three PolarGel M columns 7.5 mm × 300 mm (5 µm particle size). The columns were kept at 35 °C. The SEC system was operated under the following conditions: 0.5 mL min^−1^ flow rate, 10 μL injection volume, and 65 min run time. Data evaluation was accomplished using the ASTRA software 6.1 package.

#### 4.2.2. Lignin Polyurethane Aerogel Synthesis

Gelation: lignin polyurethane organogels were produced by adding, 1 g of catalyst (DABCO) into 25 g of solvent in a beaker, followed by 2 g of lignin (8 wt.% lignin to solvent ratio). The mixture was stirred until solubilization occurred (if possible), forming Component A. In another container, 2 g of isocyanate (Lupranat M20S, BASF Polyurethanes GmbH, Lemfoerde, Germany) was added to 25 g of solvent, forming Component B. Once both components were fully solubilized, Component A was added to Component B and stirred immediately. The reaction speed depended on the choice of lignin and gelation solvent, with times as short as 20 seconds.

Solvent Exchange and Supercritical CO_2_ Drying: the solvent in the organogel was replaced with pure ethanol (EtOH, 99.8%, Carl Roth GmbH & Co. KG, Karlsruhe, Germany) until the final ethanol concentration within the organogel substrates reached at least 97.0 wt.% (monitored by density measurements using an Anton Paar DMA 4500 M, Anton Paar GmbH, Graz, Austria). The organogels were then placed in an autoclave and dried for 4 h with supercritical CO_2_ at a flow rate of approximately 120 g min^−1^, at 120 bar and 60 °C. After depressurization at a rate of about 10 bar min^−1^, the dry samples were removed from the autoclave and stored in sealed vessels in a desiccator until further analysis.

#### 4.2.3. Lignin Polyurethane Aerogel Characterization

Gelation time: after mixing the two components, the gel point was measured, marking the transition from liquid to gel. This transition was identified through visual observation, indicated by an increase in turbidity and a noticeable color change.

##### Shrinkage and Density

Shrinkage: volumetric shrinkage from organogel to aerogel was assessed by first calculating the volumes of cylindrical slabs for both the organogel (*V_0_*) and the aerogel (*V*) using Formula (1). The volume calculations were based on the slabs’ measured diameter (r) and height (h), which were obtained using a Vernier scale. The shrinkage was then evaluated using Formula (2), comparing the initial organogel volume to the final aerogel volume.

(1) $$V=\pi r^{2}\cdot h$$

(2) $$Shrinakge=(\frac{V_{0}-V}{V_{0}})\times 100$$

Envelope density of aerogels (*ρ*_e_) was determined based on the mass and volume of the substrates:

(3) $$\rho_{e}=\frac{m}{v}$$

while mass (*m*) was determined by weighing the samples on a precision balance (Model: Precisa S520, Precisa Gravimetrics AG, Dietikon, Switzerland).

Skeletal density: the skeletal density *ρ*_s_ was determined via helium pycnometer (Multivolume Micromeritics 1305, 6-fold measurement, Micromeritics Instrument Corporation, Norcross, GA, USA).) at room temperature using a 1 cm^3^ sample holder. The overall porosity ε was estimated from the envelope and skeletal densities:

(4) $$\varepsilon =\left(1-\frac{\rho_{e}}{\rho_{s}}\right)\cdot 100\%$$

Porosity and Specific surface area (*S*_v_): the specific surface area (*S*_v_) was analyzed using nitrogen multilayer adsorption measurements, performed with a Nova 3000e Surface Area Analyzer (Quantachrome Instruments, Boynton Beach, FL, USA). Approximately 20 mg of sample was used for each test. Before analysis, the samples were degassed under vacuum at 60 °C for 4 h. The S_v_ was evaluated using the BET (Brunauer–Emmett–Teller) method, based on a linear fit of the BET model within a relative pressure (p/p_0_) range = 0.027–0.27, R^2^ ≥ 0.997) derived from Type IV N_2_ adsorption isotherms [84,85].

Scanning electron microscopy: for the characterization of the inner pore structure of the lignin polyurethane aerogels, scanning electron microscopy (Zeiss Supra VP55, Carl Zeiss AG, Jena, Germany) was used, the substrates were cut open with a razor blade and coated with a conductive, thin gold layer (approximately 10 nm) using a Sputter Coater SCD 050 (BAL-TEC, Balzers, Liechtenstein) before analysis. Measurements were conducted under a high vacuum with an accelerating voltage of 3 kV, utilizing an in-lens detector at a working distance of 4.0 mm.

FTIR Analysis: a single-bounce diamond ATR insert (MIRacle, Pike Technologies, Fitchburg, MA, USA) and a liquid-cooled MCT detector were used to measure ATR MIR spectra with the Vertex 70 spectrometer (Bruker Optik GmbH, Ettlingen, Germany). The spectrometer was purged with dry, carbon dioxide-free air. Spectra were recorded from 4000 cm^−1^ to 600 cm^−1^, with 63 scans per spectrum, using the OPUS 7.0 software (Bruker Optik GmbH, Ettlingen, Germany). Data processing was conducted using The Unscrambler X (Version 10.5, Camo Software, Oslo, Norway). Prior to analysis, the spectra underwent several pre-processing steps, including baseline correction and truncation (selecting wavenumbers within the range of 600 cm^−1^ to 4000 cm^−1^ for analysis). Area normalization was performed for comparison purposes, and a baseline offset was applied.

Mechanical tests: uniaxial compression tests were conducted using a TA.XTplusC Texture Analyzer (Stable Micro Systems, Godalming, UK) to assess the stiffness and fracture behavior of aerogel samples. The samples, with thicknesses ranging from 5.0 to 8.9 mm for DA and from 6.4 to 11.3 mm for PA, were compressed using a 4 mm diameter piston at a constant speed of 0.15 mm/s. The tests continued until either a 40% compressive strain was reached or the maximum load capacity of 500 N was achieved. All samples were measured in triplicate.

Thermal stability tests: Thermal stability of the samples was evaluated according to ASTM E1131 guidelines, “Standard Test Method for Compositional Analysis by Thermogravimetry”. Approximately 180 mg of each sample was tested using a PT1600 Linseis thermogravimetric analysis device (TGA, Linseis Messgeräte GmbH, Selb, Germany) under a nitrogen atmosphere. The samples were first dried at 80 °C for one hour, then heated to 800 °C at a rate of 5 °C/min.

## Figures and Tables

**Table 1:** Characterization of the different lignin types and their main functional groups.

Lignin Type	Source	Purification Process	Molecular Weight			Ash Content	Klason Lignin Content Minus Ash	Methoxyl Groups	Aromatic OH Groups	Total OH, Groups
			Mn	Mw	Mw/Mn (P_d_)					
			kDa			%	%	mmol g^−1^	mmol g^−1^	mmol g^−1^
Aquasolv	Wheat straw	Hydrothermal + Enzyme	2.2	4.8	2.14	10.15	70.77	2.47	1.76	2.71
Organosolv	Beech wood	Organosolv pulping	1.9	3.7	1.96	0.13	89.51	6.42	3.25	5.03
Soda	Wheat straw	Soda pulping	2.2	6.2	2.84	2.44	-	-	0.87	1.35

**Table 2:** Textural properties of lignin polyurethane samples fabricated in different solvents.

Solvent	t_gel_	Volumetric Shrinkage	$\rho_{e}$	$\rho_{s}$	ε	*S*_v_
	(seconds)	(*v*/*v*)	(g cm^−3^)	(g cm^−3^)	(%)	(m^2^ g^−1^)
Acetone	300	41.7 ± 0.91	0.12 ± 0.016	3.39 ± 0.144	96.3	0
DMSO	19	80.5 ± 0.76	0.80 ± 0.005	1.32 ± 0.002	39.0	77.2
Pyridine	139	76.0 ± 0.05	0.54 ± 0.031	1.40 ± 0.001	61.1	23.4

## Data Availability

The raw data supporting the conclusions of this article will be made available by the authors on request.

## Supplementary Materials

The following supporting information can be downloaded at https://www.mdpi.com/article/10.3390/gels10120827/s1, Table S1. Hildebrand and Hansen solubility parameters for the different solvents [45,46,47,48]; Figure S1. Solubility of Aquasolv, Soda, and organosolv lignin in different solvents at 1% (A) & 5% (B); Figure S2. (A) Derivative Thermogravimetric analysis (DTG) of pure Organosolv lignin and (B) Thermogravimetric analysis (TGA) of DMSO-based samples, measured in nitrogen at 5 K min; Figure S3. (A) Derivative Thermogravimetric Analysis (DTG) and (B) Thermogravimetric analysis (TGA) of pyridine-based samples, measured in nitrogen at 5 K min; Figure S4. Evaluation of Young’s modulus from the linear region of stress-strain curves for DMSO-based samples; Figure S5. Evaluation of Young’s modulus from the linear region of stress-strain curves for pyridine-based samples; Table S2. Summary of linear regression fit parameters used for the evaluation of Young’s modulus (in Figures S4 and S5).
