# Supplementary material for: Lignin Polyurethane Aerogels: Influence of Solvent on Textural Properties
Source: Gels. 2024 Dec 14;10(12):827. doi: 10.3390/gels10120827 (PMC11675100; doi:10.3390/gels10120827)
Supplement: Supplementary file 1 [file gels-10-00827-s001.zip › gels-3328023-supplementary.pdf]

gels

MDPI

**Supplementary Materials:** The following supporting information can be downloaded at: https://www.mdpi.com/article/10.3390/gels10120827/s1, Figure S1: title; Table S1: title; Video S1: title.

**Table S1**. Hildebrand and Hansen solubility parameters for the different solvents [45–48].

| Solvent | Hilderbrand Δ | Hansen | | |
|---|---|---|---|---|
| | | δd | δp | δh |
| | $MPa^{1/2}$ | $MPa^{1/2}$ | $MPa^{1/2}$ | $MPa^{1/2}$ |
| Water | 47.80 | 15.5 | 16.0 | 42.3 |
| Acetone | 19.94 | 15.5 | 10.4 | 7.0 |
| Methanol | 29.61 | 15.1 | 12.3 | 22.3 |
| DMSO | 26.68 | 18.4 | 16.4 | 10.2 |
| Ethanol | 26.52 | 15.8 | 8.8 | 19.4 |
| Ethylacetate | 18.15 | 15.8 | 5.3 | 7.2 |
| MEK | 19.05 | 16.0 | 9.0 | 5.1 |
| Pyridine | 10.6 | 19.0 | 8.8 | 5.9 |

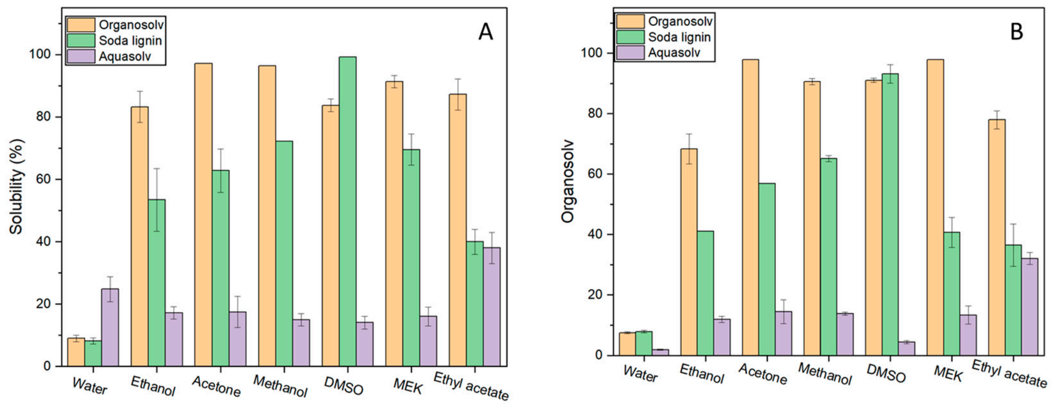


**Figure S1.** Solubility of Aquasolv, Soda and Organosolv lignin in different solvent at 1% (A) & 5% (B).

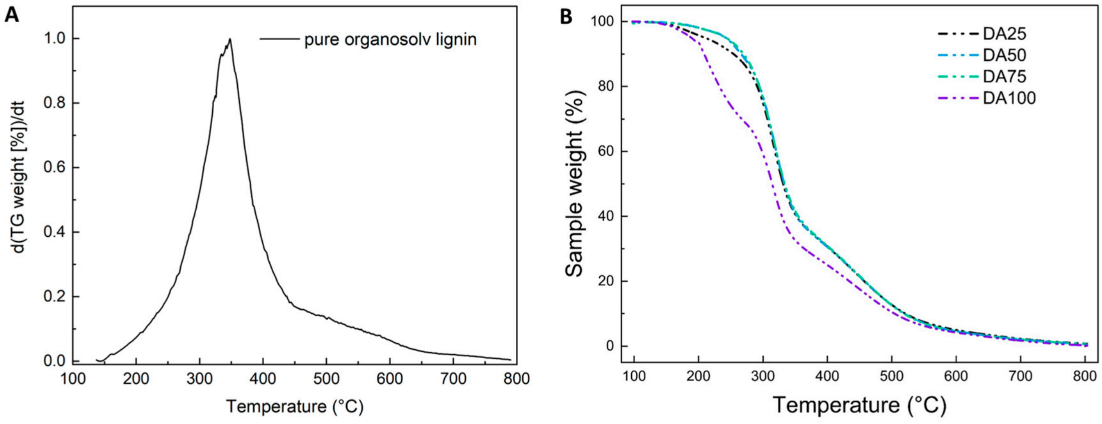

**Figure S2.** (A) Derivative Thermogravimetric analysis (DTG) of pure Organosolv lignin and (B) Thermogravimetric analysis (TGA) of DMSO based samples, measured in nitrogen at 5 K min.

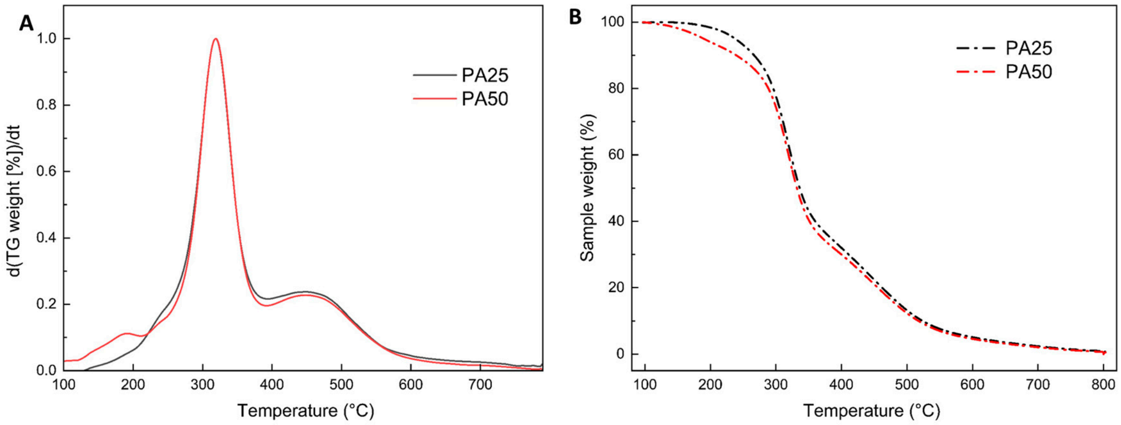


**Figure S3.** (A) Derivative Thermogravimetric Analysis (DTG) and (B) Thermogravimetric analysis (TGA) of the Organosolv lignin polyurethane aerogels produced using solvent mixtures of Pyridine/Acetone (PA).

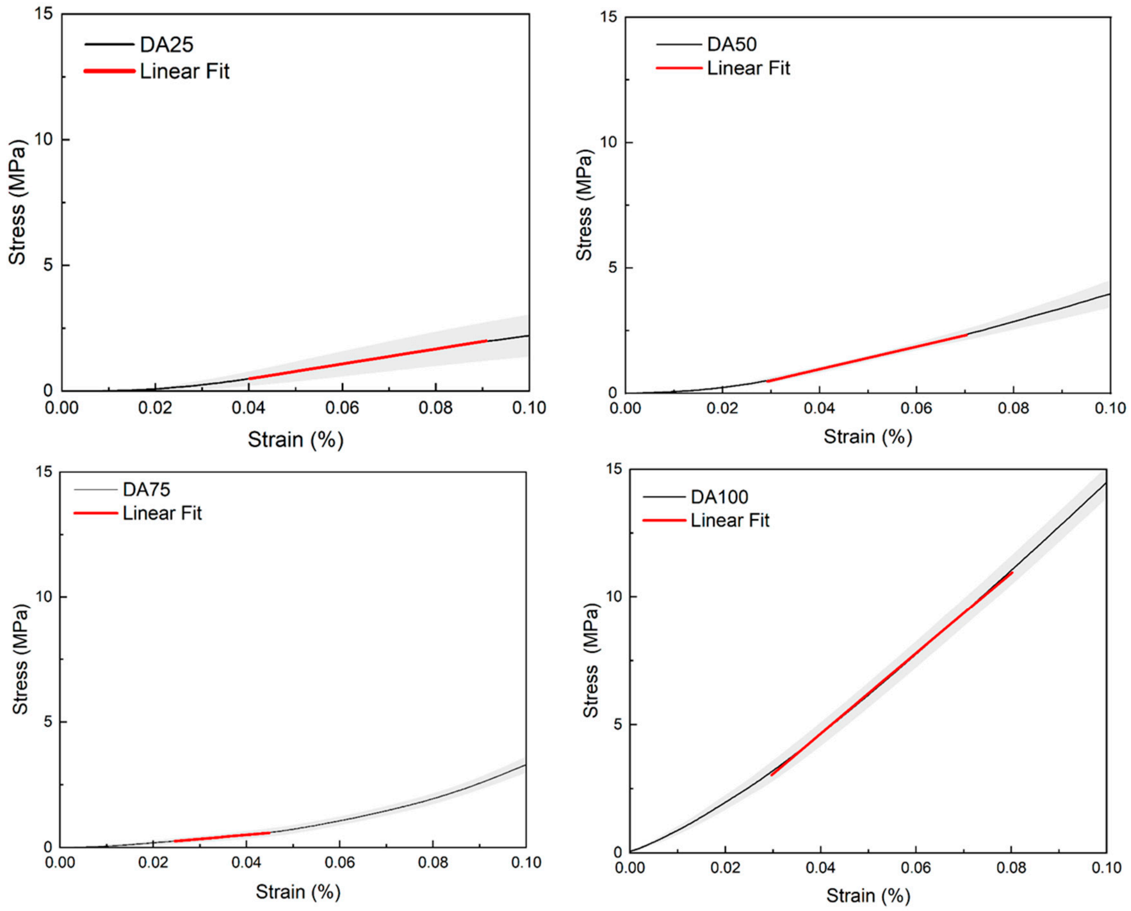


**Figure S4.** Evaluation of young's modulus from linear region of stress strain curves for DMSO based samples, samples were measured in triplicates (n=3).

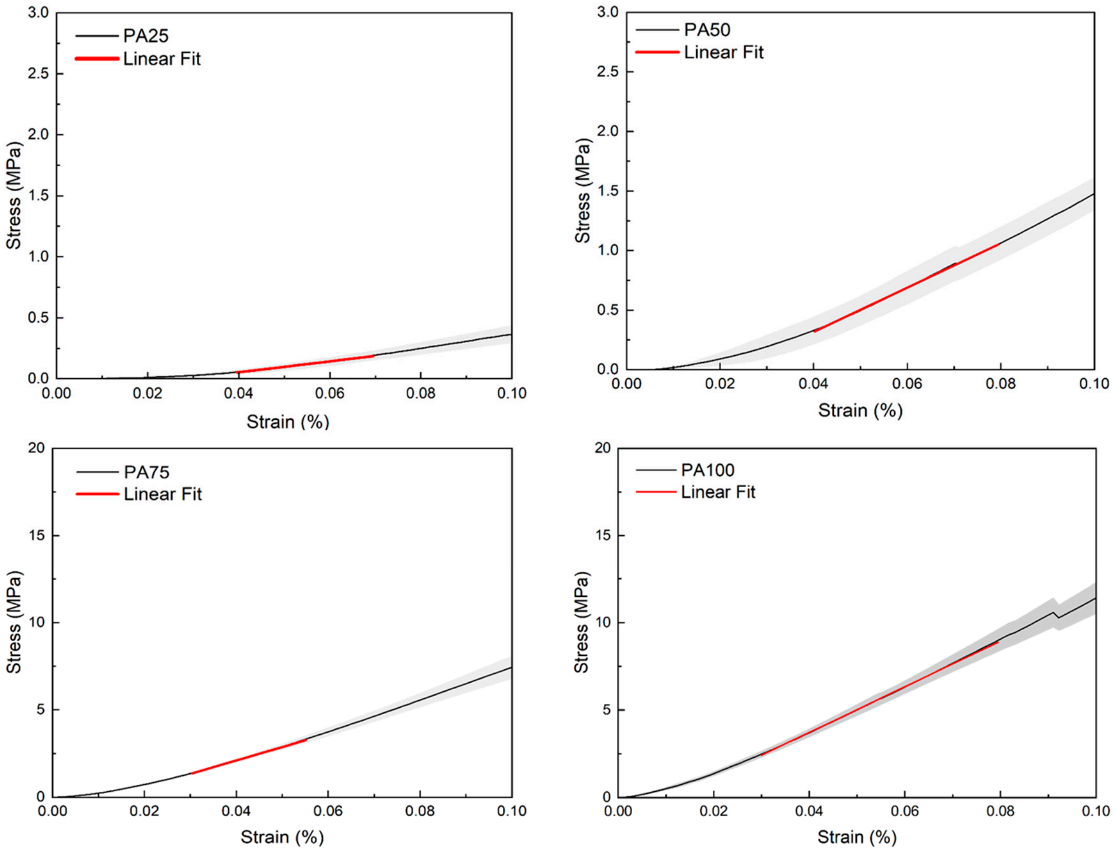


**Figure S5.** Evaluation of young's modulus from linear region of stress strain curves for Pyridine based samples, , samples were measured in triplicates (n=3).

**Table S2**. Summary of linear regression fit parameters used for the evaluation of Young's modulus (in Figure S4 & S5).

| Sample | R-Square (COD) | Adj. R-Square | Pearson's r |
|---|---|---|---|
| DA25 | 0.99983 | 0.99983 | 0.99992 |
| DA50 | 0.99897 | 0.99894 | 0.99948 |
| DA75 | 0.99598 | 0.99589 | 0.99799 |
| DA100 | 0.9994 | 0.99939 | 0.9997 |
| PA25 | 0.99563 | 0.99543 | 0.99781 |
| PA50 | 0.99931 | 0.99929 | 0.99965 |
| PA75 | 0.99886 | 0.99884 | 0.99943 |
| PA100 | 0.99962 | 0.99961 | 0.99981 |
